# *USP8* mutation in Cushing's disease

**DOI:** 10.18632/oncotarget.4856

**Published:** 2015-07-13

**Authors:** Chuanxin Huang, Yongyong Shi, Yao Zhao

**Affiliations:** Department of Neurosurgery, Huashan Hospital, Shanghai Medical College, Fudan University, Shanghai, China

Pituitary corticotroph adenomas, also referred to as Cushing's disease (CD), secret large amounts of adrenocorticotropic hormone (ACTH), resulting in excess glucocorticoids and hypercortisolism [1]. The diagnosis of hypercortisolism is complicate and sometimes difficult because its clinical features overlap with other common diseases. Currently, 65-90% of patients achieve complete or partial remission after initial transsphenoidal surgery. However, surgical clues are sometimes hindered by failure to locate the tumor. Moreover, it is particularly difficult to effectively treat patients with residual cortisol excess due to incomplete removal of tumor tissues or recurrent tumors. The genetic pathogenesis of CD remains obscure, hampering the development of novel diagnostic and therapeutic strategies for this disease.

Fortunately, two recent studies demonstrated that the ubiquitin-specific protease 8 (USP8) gene is frequently mutated in corticotroph adenomas [2,3]. One of these studies identified *USP8* mutation in 6 of 17 corticotroph adenomas [2], while the other showed that *USP8* was mutated in 75 of 120 corticotroph adenomas [3]. The later study also reported that *USP8* mutation was undetected in other pituitary adenoma types, including somatotroph adenomas and prolactin-secreting adenomas. Mutations of guanine nucleotide-binding protein subunit (*GNAS*) and the Aryl Hydrocarbon receptor interacting protein (*AIP*) gene are preferentially associated with somatotroph and prolactin-secreting adenomas [4]. These facts support the notion that the genetic origin of pituitary adenomas is heterogeneous and corticotroph adenoma is pathogenically distinct. Moreover, *USP8* mutations are rarely found in cancers (1%) based on the COMSINC and TCGA database. Collectively, *USP8* mutation is a common and specific genetic alteration in corticotroph adenoma, providing the novel insight into the molecular pathogenesis of this disease. Testing the status of *USP8* mutation may be a useful strategy for the diagnosis of CD.

All identified *USP8* mutations are located within or adjacent to the 14-3-3 binding motif (RSYSS) of the USP8 protein. USP8 is a deubiquitinase (DUB) and can be phosphorylated in its RSYSS motif, leading to association with 14-3-3 protein and subsequent impairment of its DUB activity [5]. Three identified dominant *USP8* mutants failed to bind to 14-3-3 protein and displayed elevated DUB activity through testing the epidermal growth factor receptor (EGFR) as substrate. Furthermore, these *USP8* mutations facilitate proteolytic cleavage of USP8 into two fragments, one of which processes higher DUB activity compared to full-length USP8.

**Figure 1:**
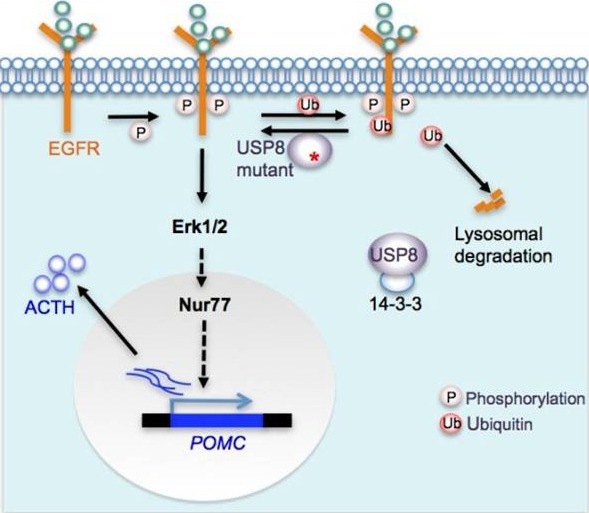
*USP8* mutation contributes to ACTH overproduction USP8 deubiquitinates EGFR and protects it from lysosomal degradation. USP8 mutants lose the binding to 14-3-3 protein, resulting in the increased DUB activity and more sustained EGFR-Erk1/2 signaling. Erk1/2 activation promotes POMC transcription probably through Nur77.

In response to EGF, EGFR is activated to trigger multiple downstream signaling pathways including MAPK, and subsequently undergoes polyubiquitination for lysosomal degradation to switch down EGFR signaling. The identified *USP8* mutants increase EGFR deubiquitination to inhibit EGF-induced EGFR downregulation, leading to augmented and more sustained EGFR signaling (Figure 1). This is supported by this fact that corticotroph adenomas harboring *USP8* mutation display a higher incidence of EGFR expression and protein abundance as well as phosphorylated Erk1/2.

*USP8* mutations contribute to ACTH overproduction in CD. *USP8*-mutated corticotroph adenomas had higher ACTH production and expressed more POMC (encoding the precursor of ACTH) mRNA than those with wild type *USP8*. *USP8* knockdown using shRNA in primary corticotroph adenoma cells effectively reduced ACTH production. Ectopic expression of *USP8* mutants or cleaved *USP8* in murine corticotroph cell line induced higher POMC promoter activity and transcription than wild-type *USP8*. Mechanistically, *USP8* mutation potentiates EGFR-Erk1/2 signaling, probably resulting in the activation of Nur77, a key transcription activator of the POMC gene (Figure 1) [6]. Among numerous USP8 substrates, EGFR appears to be the pivotal mediator for ACTH overproduction in *USP8*-mutated CD. In the absence of EGFR, USP8 was unable to augment the POMC promoter activity in luciferase reporter assay, and gefitinib, an EGFR inhibitor, significantly impaired the ACTH production in primary corticotroph adenoma cells [3,7]. Besides EGFR, USP8 may regulate other receptor tyrosine kinases and additional pathways which are expressed in corticotroph cells and control ACTH production and secretion. The identification of these USP8 targets will be helpful in understanding the mechanism of USP8 action in CD and discovering novel therapeutic targets. Although the proliferation action of *USP8* mutant is similar to that of wild-type *USP8* in corticotroph-derived cell line, it is unclear whether this was the case in *vivo* [2]. *USP8* mutation is expected to have multiple effects other than ACTH hypersecretion and may be important for corticotroph tumorigenesis, probably through accelerating the cell cycle inhibitor p27(Kip1) degradation, resulting from constitutive activation of EGFR-Erk1/2. Mice deficient in p27(Kip1) have been reported to develop ACTH-secreting pituitary adenomas. Mice with the desired *USP8* mutation in the 14-3-3 binding motif are urgent to be developed to dissect the exact roles of *USP8* mutation in the pathogenesis of corticotroph adenoma.

Surgery alone is not effective in treatment of most CD cases. Optimum treatment of patients with the recurrent tumor and/or residual tumor tissues requires the development of new drugs. Inhibiting the DUB activity of USP8 seems to be a promising therapeutic strategy for *USP8*-mutated cases. Further efforts are needed to develop the specific USP8 inhibitor for treatment of CD. Considering that EGFR plays a critical role in mediating USP8 action in corticotroph adenomas, treatment of Gefitinib seems to be a safe and effective in patients harboring *USP8*-mutated adenoma.
